# A New Blood Pulsation Simulator Platform Incorporating Cardiovascular Physiology for Evaluating Radial Pulse Waveform

**DOI:** 10.1155/2019/4938063

**Published:** 2019-02-11

**Authors:** Tae-Heon Yang, Jaeuk U. Kim, Young-Min Kim, Jeong-Hoi Koo, Sam-Yong Woo

**Affiliations:** ^1^Department of Electronic Engineering, Korea National University of Transportation, Chungju-si 27469, Republic of Korea; ^2^Future Medicine Division, Korea Institute of Oriental Medicine, Daejeon 34054, Republic of Korea; ^3^Department of Mechanical and Manufacturing Engineering, Miami University, Oxford 45056, Ohio, USA; ^4^Center for Mechanical Metrology, Korea Research Institute of Standards and Science, Daejeon 34113, Republic of Korea

## Abstract

To meet the need for “standard” testing system for wearable blood pressure sensors, this study intends to develop a new radial pulsation simulator that can generate age-dependent reference radial artery pressure waveforms reflecting the physiological characteristics of human cardiovascular system. To closely duplicate a human cardiovascular system, the proposed simulator consists of a left ventricle simulation module, an aorta simulation module, a peripheral resistance simulation module, and a positive/negative pressure control reservoir module. Simulating physiologies of blood pressure, the compliance chamber in the simulator can control arterial stiffness to produce age-dependent pressure waveforms. The augmentation index was used to assess the pressure waveforms generated by the simulator. The test results show that the simulator can generate and control radial pressure waveforms similar to human pulse signals consisting of early systolic pressure, late systolic pressure, and dicrotic notch. Furthermore, the simulator's left ventricular pressure-volume loop results demonstrate that the simulator exhibits mechanical characteristics of the human cardiovascular system. The proposed device can be effectively used as a “standard” radial artery pressure simulator to calibrate the wearable sensor's measurement characteristics and to develop more advanced sensors. The simulator is intended to serve as a platform for the development, performance verification, and calibration of wearable blood pressure sensors. It will contribute to the advancement of the wearable blood pressure sensor technology, which enables real-time monitoring of users' radial artery pressure waveforms and eventually predicting cardiovascular diseases.

## 1. Introduction

The importance of real-time monitoring a radial artery pressure and its waveform is steadily increased in the medical science and healthcare field. This is because the radial pulsation is a surrogate maker to estimate a central aortic pressure and its waveform which is clinically imperative for predicting cardiovascular diseases [1–4]. Currently, there exist numerous radial blood pressure measuring devices in the market, and the recent trend is to develop wearable devices, which allow real-time monitoring of radial pressure waveforms.

One of the most common commercially available radial blood pressure monitoring devices is cuff-type wrist blood pressure monitors. They can measure diastolic and systolic radial artery pressure relatively easily, but they pressurize the wearer's wrist with the cuff at the time of measurement using the oscillometric method, which gives significant inconvenience to the users in real-time measurement [5]. More importantly, the cuff-type pressure monitors cannot measure the pressure waveforms of radial artery which is essential for cardiovascular disease prediction. In order to monitor the radial artery of the wrist in real time economically and simply, the optical technique of photoplethysmography (PPG) has been widely studied [6]. The PPG is a noninvasive optical technique that detects microvascular blood volume changes in tissues, and it is currently applied to commercial products, such as Samsung Galaxy Gear and Apple Watch [7, 8]. It has the possibility to measure blood pressure and waveforms through an extension to the imaging PPG (IPPG) technology by adopting an additional camera [9]. Nonetheless, in order to measure both the amplitude and the waveform of blood pressure precisely, such portable health monitoring devices that incorporate the PPG or the IPPG technologies have to be validated through costly and complex clinical trials. Due to these limitations and constraints, they are currently used only for monitoring human's heart rate.

Referring to another drawback of PPG, the PPG sensor should be in direct contact with the skin during measurement. However, it is quite challenging to maintain the contact between the sensor and the skin at the interface of the PPG watch or mobile device. To overcome this shortcoming, there have been numerous research studies on flexible and wearable sensing technologies aiming at developing a skin-attachable blood pressure sensor with superior sensing properties along with mechanical flexibility and robustness, enabling real-time measurements of blood pulses. Recently, various nanomaterials including nanowires [10], carbon nanotubes [11], polymer nanofibers [12], metal nanoparticles [13], and graphene [14] have been used for the design of novel, flexible pressure sensors.

In light of the recent surge of flexible and wearable pressure sensor research and development for measurement of blood pressure and pulse waveforms, there exists an increasing need for securing the accuracy of blood pressure measurement devices. The accuracy of blood pressure measurement is critically important for the commercial use of such wearable sensors. In the case of hypertension, which is the main concern of blood pressure measurement, it is known that a 5 mmHg error in blood pressure measurements may double the number of patients diagnosed with hypertension, or even reduce it by half [15]. Despite the importance of measurement accuracy, little studies exist on the evaluation and improvement of the measurement accuracy of wearable blood pressure sensors.

While a clinical testing involving a large number of human subjects is the best way to study the output accuracy of the wearable monitoring sensors, it is not desirable. This is because such clinical testing is costly and time consuming. Moreover, it has its own challenges as it has to account for variance of human race, sex, age, and health Conditions. Alternative to clinical testing, this study proposes to develop a radial artery waveform simulator, which can be served as a platform for evaluating wearable blood pressure sensors. The simulator should be capable of producing the standardized radial artery pressure waveforms (replicating radial pulse waveforms generated in the terminal reason of radial arteries in human wrists) and controlling the pressure waveforms according to various factors, such as ages. This kind of simulator can dramatically reduce the cost and the development life cycle of the wearable blood pressure sensors. Moreover, it can be used as a sensor calibration system for mass production of the wearable sensors. In short, a pulse waveform simulator can greatly contribute to the commercialization of the wearable pressure sensor technology.

Unlike few existing cardiovascular simulators which used for the in vitro test of artificial organs and the evaluation of heart assist device performance [16–18], in this study, a radial pulsation simulator is designed and constructed that is capable of producing variable shape of arterial pressure waveforms. The function of existing simulators is limited to simply creating the circulation of the fluid. Therefore, the main goal of this study is to develop a new simulator that can generate radial artery pressure waveforms reflecting the physiological characteristics of the human's cardiovascular system for the evaluation of wearable blood pressure sensors.

This article is organized as follows. The next section describes the target pulse pressure waveform of the radial artery that the proposed simulator is trying to reproduce. The following design and development section explains the schematic diagram and the developed platform of the radial pulsation simulator based on the physiological behaviour of the human body. Finally, after describing the process of the pressure data measurement from the developed simulator, analysis and discussion of experimental results conclude the article.

## 2. Human's Radial Artery Pressure Waveform

The primary objective of the proposed radial pulsation simulator is to reproduce and control the standardized radial artery pressure waveforms generated at a human's wrist for evaluating the wearable pressure sensors. For this purpose, it is necessary to clarify the target pulse pressure waveform of the radial artery for being reproduced in the simulator and closely analyze the physiological factors that determine the shape of the waveform.

**Figure 1:**
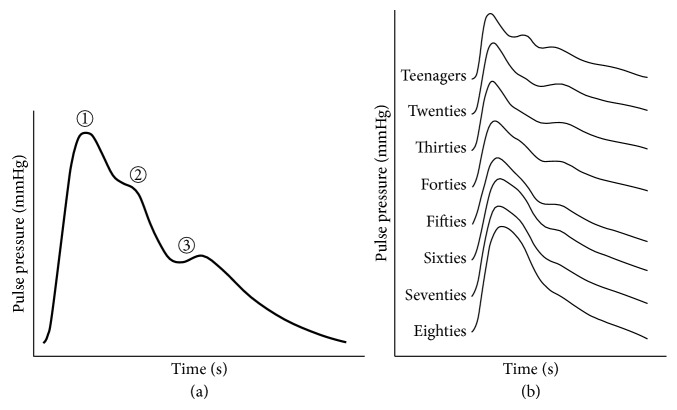
A pulse pressure waveform of a radial artery: (a) the averaged pulse pressure waveform of the forties; (b) the averaged pulse pressure waveform from the teenagers to the eighties.

Figure 1(a) shows the averaged radial pulse pressure waveform of the forties [19]. In an idealized model of the arterial system, the early systolic pressure (①) is created by the forward propagated pressure wave due to the rapid contraction of a left ventricle [20]. This pressure wave is propagated through the thoracic aorta and it encounters the stagnant blood at the abdominal aortic bifurcation and produces a reflected wave in the opposite direction to the blood flow [20]. The reflected wave summates with the forward wave to establish the late systolic pressure shoulder (②). The shoulder begins to diminish until the pressure wave reaches the second shoulder (③) marked by the closure of the aortic valve with consequent rebound of blood. Figure 1(b) demonstrates the averaged radial pulse pressure waveforms from the teenagers to the eighties [19]. The radial pulse contour in children shows multiple prominent fluctuations. With advancing age, these become less distinct and the systolic peaks progressively broaden, although the maximum still usually occurs in early systole. This tendency is due to the increase in the pulse wave velocity (PWV) and the magnitude of the reflected wave, which are affected by the increase in arterial stiffness with age [3]. The change in arterial stiffness is a main factor for changing the properties of the reflected wave which is important in blood pressure monitoring. Therefore, it has been concluded that it is essential to develop the radial pulsation simulator with the function for adjusting the arterial stiffness, and it allows the developed simulator to generate various blood pressure waveforms associated with cardiovascular events.

## 3. A Radial Pulsation Simulator

**Figure 2:**
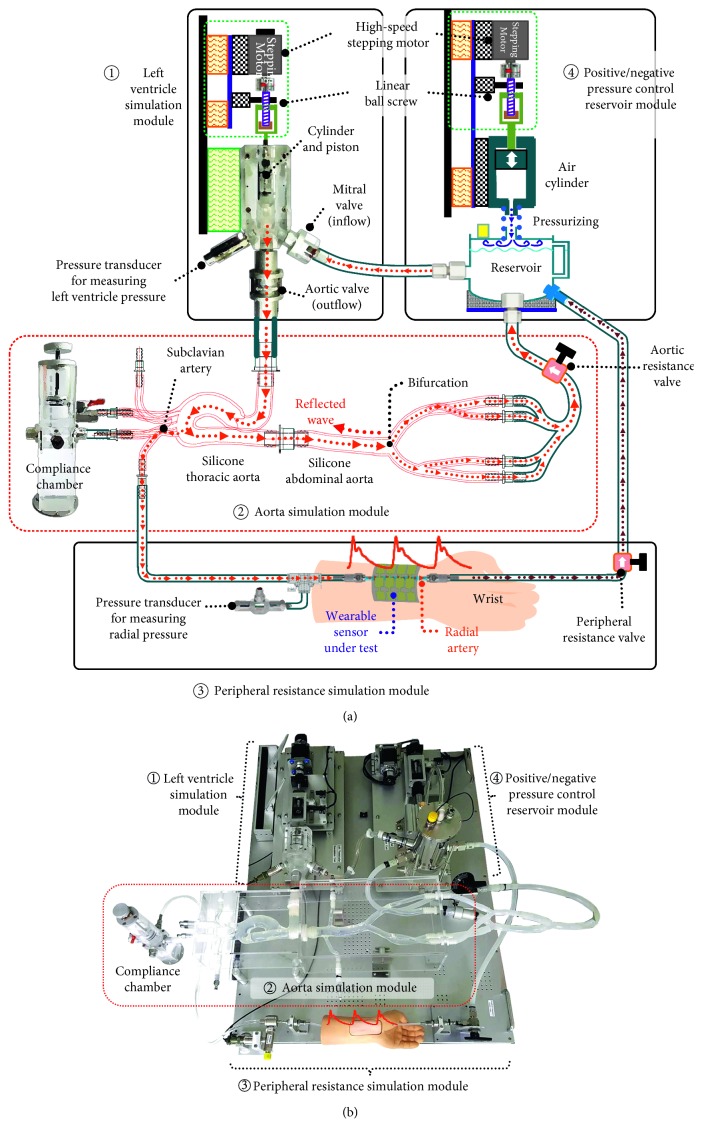
A new radial pulsation simulator: (a) schematic diagram; (b) developed system.

In terms of the physiological point of view, while the diastolic blood pressure is determined by peripheral vascular resistance, the systolic pressure wave is directly decided by left ventricular ejection, arterial stiffness, and pulse wave reflection. As shown in Figure 2, the simulator is composed of left ventricle simulation module (①), aorta simulation module (②), peripheral resistance simulation module (③), and positive/negative pressure control reservoir module (④). The pressure control reservoir is specifically designed to adopt “human-alike” simulator that can imitate the radial arterial pressure waveform generated by physiological characteristic of human's cardiovascular system.

Figure 2(a) shows a conceptual diagram of the proposed radial pulsation simulator, and it shows the pathway of fluid in the simulator. By pushing the piston with a linear motor, the fluid is released from the left ventricle simulation module to the aorta simulation module as shown in ①. The pressure wave of the released fluid will contain reflected waves by reaching the stagnant fluid at the abdominal aorta intersection, and then subsequently the amplitude of the fluid's pressure wave is buffered and regulated in a compliance chamber as demonstrated in ②. The partial fluid will be recollected into the reservoir while the other fractional fluid initially moves to the peripheral resistance simulation module which causes to release pressure wave on the wrist silicon radial artery and then is recollected to the reservoir (②–④ in Figure 2). The fluid collected in the reservoir is injected into the left ventricle cylinder again when a piston moves backward. Consequently, the fluid keeps flowing in the circulation of system.

Figure 2(b) shows the developed prototype of the radial pulsation simulator based on the physiological mechanism explained above. In order for the simulator to be “human-alike,” it is essential to design the system that can produce analogous pulsation with actual cardiac output. Thus, as shown in ① of Figure 2(b), the left ventricle simulation part is designed to include a motor system which reciprocates the piston with specific radius and length in the cylinder to release maximum 80 mL of fluid every 750 ms. In order to prevent the residual vibration generated by the high-speed reciprocating motion of the piston from being transmitted to the fluid and creating noise signal in the pressure waveform of the fluid, the reciprocating motor system was developed and mounted on a separate base with damping pads in the vertical and horizontal directions. The acrylic cylinder is manufactured to be equipped with a mitral check valve (fluid inflow), an aortic check valve (fluid outflow), and a pressure transducer to monitor the pressure inside the cylinder, called left ventricular pressure. The mitral check valve was developed to control the inflow of the fluid by moving the membrane so that the opening pressure is as small as 14 mmHg, similar to the pressure in human body [21]. The aortic check valve is configured to employ a streamlined plunger for minimizing turbulence and pressure drop when fluid is released, and the opening pressure is designed to be approximately 100 mmHg, similar to the human body [22]. The fluid released from the aortic check valve flows into the aorta simulation module (② in Figure 2(b)), which is composed of an artificial silicone thoracic aorta installed with the compliance chamber and an artificial silicone abdominal aorta with bifurcation for generating a reflective wave. As the fluid initially releases, the incident wave occurs and collides with the fluid held in the bifurcation of the abdominal aorta which causes the formation of the reflected wave. This reflected wave is aggregated with the incident wave to form a central aortic pressure wave which is buffered in the compliance chamber acting as an air spring. The compliance chamber uses a piston to alter air volume in the chamber in order to regulate the arterial stiffness of the aorta simulation module. The central pressure wave including the generated reflected wave is transmitted through the subclavian artery to the radial artery of the peripheral resistance simulation module (③) to generate the radial artery pressure wave and then return to the positive and negative pressure control reservoir module (④). The reservoir module (④) is structured with a reservoir and an air pressure regulating module consisting of an air cylinder and a linear actuating motor system. The air cylinder is driven by the linear actuating motor system to regulate the air pressure in the reservoir so that the diastolic pressure in the aorta and radial artery can be 80 mmHg, the same as the pressure in human body.

In summary, this simulator is designed to generate and control the pulsating blood pressure waveforms including reflected waves in a range between diastolic and systolic pressure based on the principle similar to the cardiovascular mechanism of the human body. Hence, the simulator can generate and control the radial artery waveform reflecting the cardiovascular condition like a human body near the wrist. The systolic pressure of this pressure waveform can be regulated by controlling the motor of the left ventricle simulation module (①), and the diastolic pressure can be adjusted by operating the air cylinder of the negative pressure control reservoir module (④). In addition, the shape of the pressure waveform mainly influenced by the reflected wave can be manipulated by operating the compliance chamber of the aorta simulation module (②). Thus, the simulator can generate a pressure waveform similar to the human radial artery waveform near the wrist (③ in Figure 2), and the simulator can be used as a standard device to evaluate and improve the measurement accuracy of the various wearable blood pressure (BP) sensors by attaching it to the simulator's wrist (③ in Figure 2(a)).

## 4. Experimental Results and Discussions

The main purpose of developing a simulator is to generate and control the radial artery pressure waveform reflecting the physiological characteristics of the human's cardiovascular system. As the target pressure waveform is generated at the radial artery of the peripheral resistance simulation module (③ in Figure 2), the pressure waveform in the radial artery was measured and evaluated using a precise pressure transducer installed at the beginning part of the radial artery.

**Figure 3:**
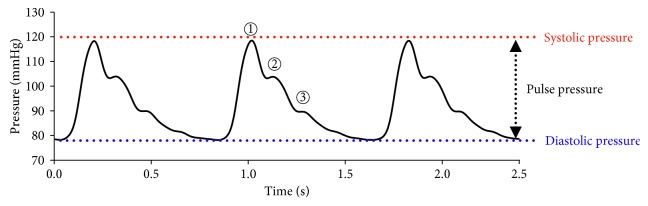
Experimental results of radial artery pressure waves generated by the simulator.

Figure 3 shows the experimental measurement results of radial artery pressure waveforms generated by the developed simulator. The pressure waveforms generated from the simulator have a similar pattern as those measured actual human data; early systolic pressure (①), late systolic pressure (②) and dicrotic notch (③). In order to regulate radial artery pressure waveforms similar to the ones from human body, three pressure adjusting mechanisms are incorporated in the simulator. First of all, the air cylinder of the reservoir (④ in Figure 2) can adjust the diastolic pressure up to 80 mmHg. Secondly, the aortic/peripheral resistance valves (② and ③ in Figure 2) can alter the pulse pressure up to 40 mmHg. Lastly, the stepping motor of the left ventricle simulation module can control the pumping cycle. For the testing, it is set to reciprocate at 750 ms, which is close to the average human heart rate. Generated pressure waveforms demonstrated in Figure 3 indicate that the simulator is capable of generating human blood pressure waveforms precisely.

**Figure 4:**
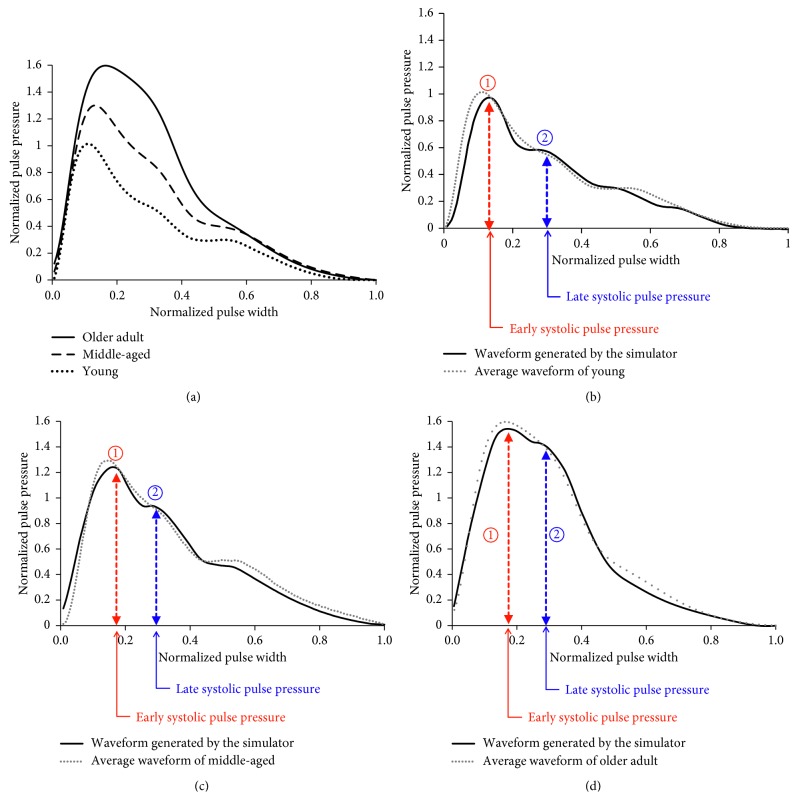
Normalized comparison between the average human's radial blood pressure waveforms and the waveforms generated by the developed simulator: (a) the average radial artery blood pressure waveforms of young, middle-aged, and older adults; (b) waveform generated by the simulator and comparison with the waveform of the young; (c) waveform generated by the simulator and comparison with the waveform of the middle-aged; (d) waveform generated by the simulator and comparison with the waveform of the older adults.

The artificial silicone thoracic aorta (② in Figure 2) of the simulator is equipped with a compliance chamber, which can control arterial stiffness by regulating the volume of air in the chamber. The piston installed vertically in the compliance chamber unit that can regulate the volume of air beyond the fluid stored in the chamber up to 10 ml, effectively making the chamber act as an air spring. The effects of changing the volume of air spring are shown in Figures 4(a) and 4(b). When the volume of air spring decreases, the late systolic pressure (② in Figure 4(b)) relatively increased more than early systolic pressure (① in Figure 4(b)). These results are consistent with those of Kohara et al. where the radial augmentation index (AI) was used to describe the results [3]. The AI is defined as equation (1), and it is widely used to clinically examine the arterial stiffness. As a human being ages, the radial augmentation index increases [3].

(1) $$\begin{array}{c} \text{Radial}\;\;\text{augmentation}\;\;\text{index}\left(\text{AI}\right)=\frac{\text{late}\;\;\text{systolic}\;\;\text{pulse}\;\;\text{pressure}}{\text{early}\;\;\text{systolic}\;\;\text{pulse}\;\;\text{pressure}}\times 100\left(\%\right), \end{array}$$

Figure 4(a) shows the mean radial artery blood pressure waveforms of young, middle-aged, and older adults. The robotic tonometry system, developed by Korea Institute of Oriental Medicine, was used to acquire the radial pulse waveforms [23–25]. Hundreds of subjects in each age group participated in radial artery blood pressure measurements. The radial artery blood pressure waveforms collected in each age group were used to generate each mean blood pressure waveform by a mathematical method (Figure 4(a)). In Figure 4(a), as age increased, radial AI and pulse pressure tended to increase, and the rates of increase were consistent with the existing literature [3, 19]. To evaluate whether the developed simulator could reproduce radial artery blood pressure waveforms of the young, middle-aged, and older adult groups, radial blood pressure waveforms were collected from the simulator while varying the volume of the air chamber with the function of controlling the degree of arterial stiffness as shown in Figures 4(b)–4(d). Figure 4(b) is the comparison between the pressure waveform generated in the developed simulator with the volume of air spring adjusted to the maximum (about 20 ml) and the average waveform of the young from Figure 4(a).  Figure 4(c) compares the normalized pressure waveforms between the generated data by the simulator and the average waveform data of the people in the middle-aged from Figure 4(a). In this case, the simulator data were collected after the volume of air spring in the compliance chamber is set to its medium value (about 10 ml). After minimizing the volume of the air spring in the chamber (about 0 ml), the normalized pressure waveform obtained from the simulator and the average waveform of the older adult were compared as shown in Figure 4(d).

In the pressure waveforms obtained from the simulator as shown in Figures 4(b)–4(d), the radial AI, defined as ② divided by ①, was calculated as 64%, 75%, and 91%, respectively, while varying the air spring volume of the chamber from maximum to medium to minimum. The radial AI obtained for each age group agrees well with those of the actual human radial artery pressure waveforms presented in previous research studies [3]. The AI results indicate that the developed simulator can control the arterial stiffness by controlling the volume of the air spring in the compliance chamber. In addition, the pulse pressures of the waveform reproduced by the simulator agree well with the human's average waveform. These results suggest that the simulator is capable generating age-dependent human pulse waveforms.

**Figure 5:**
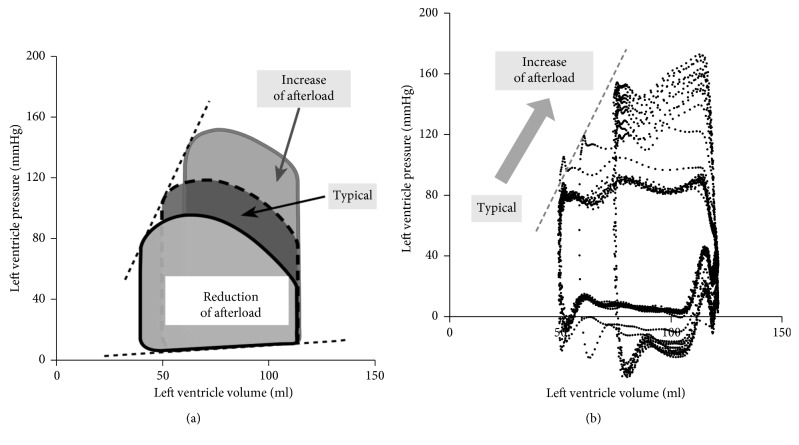
Left ventricular pressure-volume loops: (a) typical effects of afterload; (b) experimental results of measured pressure with the increase of the afterload in the developed simulator.

In addition to generating various pulse waveforms, in order for the simulator to be “human-alike,” it is required that the left ventricular pressure of the simulator (the inner pressure of the cylinder, ① in Figure 2) should be responsively changed as the actual heart when adjusting the opening amount of the aortic and peripheral resistance valves (② and ④ in Figure 2) for altering the pulse pressure. A pressure transducer mounted on the left ventricle cylinder (① in Figure 2) was used to investigate pressure-volume loops characteristics of the simulator. Generally, increasing an afterload by raising the aortic pressure will result in a smaller stroke volume and the increase of systolic pressure, as shown in Figure 5(a) [26]. On the contrary, reducing afterload by decreasing the aortic pressure will cause a larger stroke volume and the decrease of systolic pressure (Figure 5(a)) [26]. When reducing the amount of opening of the valves, the flow resistance between the arteries (② and ③ in Figure 2) and the reservoir (④ of Figure 2) increases. An increase in flow resistance has the effect of increasing afterload. The results obtained by the pressure transducer shown in Figure 5(b) indicate that the measured stroke volume and systolic pressure present similar patterns with typical human data (Figure 5(a)).

In summary, the results demonstrate that the radial simulator developed in this study can effectively reflect key characteristics of humans' cardiovascular system. The simulator is capable of generating and controlling blood pressure waveforms, so it can be efficiently used to evaluate and calibrate the measurement capability of various advanced wearable blood pressure sensors.

## 5. Conclusion

In this study, a new radial pulsation simulator capable of replicating the physical phenomenon of an actual human cardiovascular system was developed. Its performance was evaluated from the physiological perspective. The test results show that the developed simulator can be effectively used for the verification and development of wearable blood pressure sensors. Moreover, the simulator can be used to study the characteristics of cuff-type wrist blood pressure monitors to enhance the blood pressure measurement accuracy of the wrist type. Beyond the use of the simulator for blood pressure measurement technology, it is expected to contribute to the scientific advancement of western medicine and oriental medicine. For the western medicine area, the simulator replicating human cardiovascular system can replace clinical testing in some extent, substantially reducing the cost and time for cardiovascular research. In another aspect, the simulator can contribute to the modernization of oriental medicine (OM). Oriental medicine is long-established traditional medical practices in East Asia. Some OM practices include pulse diagnosis, acupuncture, and herbal medicine. The pulse diagnosis is the most important diagnostic method in OM, and it is the three figure technique that OM doctors “measure” radial pulses on the wrist by putting three fingers. However, it is an ambiguous method because it relies on OM doctors' subjective experiences. To standardize or quantify the pulse diagnosis technique, there is an urgent need for an apparatus that can generate various standard radial pulse wave patterns, which can be used to train pulse training for medical professionals. Since the simulator can generate controllable radial pressure waveforms based on the principle of cardiovascular system, it has a great potential for modernizing OM. The simulator can be used to generate various standard pulse waveforms based on cardiovascular conditions and as a training tool that a trainee can learn pulse diagnosis technique by feeling pulsations in the simulator's wrist part.

## Data Availability

The video data used to support the findings of this study are included within the supplementary information files. The video shows simple working mechanism. In the video, data are not relevant to data collection.

## Supplementary Materials

Supplementary Materials This supplementary material is a movie clip that accurately shows the operation principle of the developed radial pulsation simulator composed of left ventricular, aortic, left atrial, and left arm modules.

## References

[1] Nelson M. R., Stepanek J., Cevette M., Covalciuc M., Hurst R. T., Tajik A. J. (2010). Noninvasive measurement of central vascular pressures with arterial tonometry: clinical revival of the pulse pressure waveform?. *Mayo Clinic Proceedings*.

[2] Pauca A. L., O’Rourke M. F., Kon N. D. (2001). Prospective evaluation of a method for estimating ascending aortic pressure from the radial artery pressure waveform. *Hypertension*.

[3] Kohara K., Tabara Y., Oshiumi A., Miyawaki Y., Kobayashi T., Miki T. (2005). Radial augmentation index: a useful and easily obtainable parameter for vascular aging. *American Journal of Hypertension*.

[4] Takazawa K., Kobayashi H., Shindo N., Tanaka N., Yamashina A. (2007). Relationship between radial and central arterial pulse wave and evaluation of central aortic pressure using the radial arterial pulse wave. *Hypertension Research*.

[5] https://omronhealthcare.com/blood-pressure/wrist/

[6] Allen J. (2007). Photoplethysmography and its application in clinical physiological measurement. *Physiol. Meas*.

[7] http://www.samsung.com/us/mobile/wearables/smartwatches/gear-s3-frontier--at-t--sm-r765adaaatt/#benefits

[8] https://support.apple.com/en-us/HT204666

[9] Sun Y., Thakor N. (2016). Photoplethysmography revisited: from contact to noncontact, from point to imaging. *IEEE Transactions on Biomedical Engineering*.

[10] Takei K., Takahashi T., Ho J. C. (2010). Nanowire active-matrix circuitry for low-voltage macroscale artificial skin. *Nature Materials*.

[11] Lipomi D. J., Vosgueritchian M., Benjamin C.-K. T. (2011). Skin-like pressure and strain sensors based on transparent elastic films of carbon nanotubes. *Nature Nanotechnology*.

[12] Gao Q., Meguro H., Okamoto S., Kimura M. (2012). Flexible tactile sensor using the reversible deformation of poly (3-hexylthiophene) nanofiber assemblies. *Langmuir*.

[13] Maheshwari V., Saraf R. F. (2006). High-resolution thin-film device to sense texture by touch. *Science*.

[14] Yao H. B., Ge J., Wang C.-F. (2013). A flexible and highly pressure-sensitive graphene-polyurethane sponge based on fractured microstructure design. *Advanced Materials*.

[15] Turner M., Speechly C., Bignell N. (2007). Sphygmomanometer calibration—why, how and how often?. *Australian Family Physician*.

[16] Legendre D., Fonseca J., Andrade A. (2008). Mock circulatory system for the evaluation of left ventricular assist devices, endoluminal prostheses, and vascular diseases. *Artificial Organs*.

[17] Yokoyama Y., Kawaguchi O., Shinshi T., Steinseifer U., Takatani S. (2010). A new pulse duplicator with a passive fill ventricle for analysis of cardiac dynamics. *Journal of Artificial Organs*.

[18] Brum J., Bia D., Benech N., Balay G., Armentano R., Negreira C. (2010). Set up of a cardiovascular simulator: application to the evaluation of the dynamical behavior of atheroma plaques in human arteries. *Physics Procedia*.

[19] Kelly R., Hayward C., Avolio A., O’Rourke M. (1989). Noninvasive determination of age-related changes in the human arterial pulse. *Circulation*.

[20] SHIRWANY Najeeb A., ZOU M.-h. (2010). Arterial stiffness: a brief review. *Acta Pharmacologica Sinica*.

[21] Cheng C.-P., Igarashi Y., Little W. C. (1992). Mechanism of augmented rate of left ventricular filling during exercise. *Circulation Research*.

[22] Klabunde R. E. (2005). *Cardiovascular Physiology Concepts*.

[23] Kim Y.-M. (2017). Precise measurement method of radial artery pulse waveform using robotic applanation tonometry sensor. *Journal of Sensor Science and Technology*.

[24] Bae J.-H., Jeon Y. J., Lee S., Kim J. U. A feasibility study on age-related factors of wrist pulse using principal component analysis.

[25] Bae J.-H., Jeon Y. J., Kim J. Y., Kim J. U. (2013). New assessment model of pulse depth based on sensor displacement in pulse diagnostic devices. *Evidence-Based Complementary and Alternative Medicine*.

[26] http://www.cvphysiology.com/Cardiac%20Function/CF025

